# An open-label, single-dose study to evaluate the safety, tolerability, pharmacokinetics, and pharmacodynamics of cinacalcet in pediatric subjects aged 28 days to < 6 years with chronic kidney disease receiving dialysis

**DOI:** 10.1007/s00467-018-4054-8

**Published:** 2018-08-23

**Authors:** Winnie Y. Sohn, Anthony A. Portale, Isidro B. Salusky, Hao Zhang, Lucy L. Yan, Bella Ertik, Shahnaz Shahinfar, Edward Lee, Bastian Dehmel, Bradley A. Warady

**Affiliations:** 1 Amgen, Inc., One Amgen Center Dr. M/S 38-4-A, Thousand Oaks, CA 91320 USA; 2 UCSF Benioff Children’s Hospital, San Francisco, CA USA; 3 David Geffen School of Medicine at UCLA, Los Angeles, CA USA; 4 S. Shahinfar Consulting and Childrens Hospital of Philadelphia, Philadelphia, PA USA; 5 Children’s Mercy Hospital, Kansas City, MO USA

**Keywords:** Calcimimetics, Cinacalcet, Chronic kidney disease, Parathyroid hormone, Pediatric dialysis patients, Secondary hyperparathyroidism

## Abstract

**Background:**

Calcimimetics, shown to control biochemical parameters of secondary hyperparathyroidism (SHPT), have well-established safety and pharmacokinetic profiles in adult end-stage renal disease subjects treated with dialysis; however, such studies are limited in pediatric subjects.

**Methods:**

In this study, the safety, tolerability, pharmacokinetics (PK), and pharmacodynamics (PD) of cinacalcet were evaluated in children with chronic kidney disease (CKD) and SHPT receiving dialysis. Twelve subjects received a single dose of cinacalcet (0.25 mg/kg) orally or by nasogastric or gastric tube. Subjects were randomized to one of two parathyroid hormone (PTH) and serum calcium sampling sequences: [(1) 2, 8, 48 h; or (2) 2, 12, 48 h] and assessed for 72 h after dosing.

**Results:**

Median plasma cinacalcet *t*_max_ was 1 h (range 0.5–4.0 h); mean (SD) *C*_max_ and AUC_last_ were 2.83 (1.98) ng/mL and 11.8 (8.74) h*ng/mL, respectively; mean (SD) half-life (*t*_1/2_) was 3.70 (2.57) h. Dose adjustments, based upon body weight (mg/kg), minimized the effects of age, body weight, body surface area, and body mass index on cinacalcet PK. Reductions in serum PTH levels from baseline were observed at 2 to 8 h post-dose (median 10.8 and 29.6%, respectively), returned towards baseline by 12–72 h and were inversely related to changes in the plasma cinacalcet PK profile. Single-dose cinacalcet was well-tolerated with no unexpected safety findings and a PK/PD, safety profile similar to adults.

**Conclusions:**

In conclusion, a single 0.25 mg/kg dose of cinacalcet was evaluated to be a safe starting dose in these children aged < 6 years.

## Introduction

Chronic kidney disease-mineral and bone disorder (CKD-MBD) is a systemic disorder of mineral and bone metabolism due to CKD manifested by abnormalities of calcium, phosphorus, parathyroid hormone (PTH), or vitamin D metabolism; abnormalities of bone turnover, mineralization, volume, linear growth, or strength; and vascular or other soft tissue calcification [1, 2]. Secondary hyperparathyroidism (SHPT) develops in the course of CKD, and its prevalence and severity increase as CKD progresses towards end-stage renal disease (ESRD) [3–7]. Secondary hyperparathyroidism is associated with adverse outcomes including cardiovascular complications, vascular calcification, heart failure, calciphylaxis, bone pain, bone deformities, and increased risk of fracture [7–11]. The long-term consequences of CKD-MBD on the growing skeleton are characterized by changes in the cardiovascular system, bone, disordered skeletal development, and growth impairment [12–15]. The overall mortality rate in children with ESRD is 30-fold greater than in their healthy peers, with a large proportion of the mortality due to cardiovascular disease (CVD) [16].

Active vitamin D (1,25(OH)_2_D_3_), vitamin D analogs, and phosphate binders are the most common treatments for SHPT; however, such therapies may not fully treat SHPT and have the potential to increase the risk of vascular calcification by increasing serum calcium and phosphorus levels [17]. In addition, fracture risk, bone deformities, and growth retardation [9] may not adequately respond to active vitamin D analogs given to suppress SHPT [18], and a bone mineralization defect is persistent despite therapy with active vitamin D sterols [18]. Finally, whereas parathyroidectomy has been conducted to treat unresponsive SHPT, children (≤ 17 years of age) have significantly higher general and endocrine-specific complication rates after parathyroidectomy than adults [19, 20]. Interestingly, children aged ≤ 6 years have been found to have significantly longer hospital stays and higher complication rates than children aged 7–12 years and 13–17 years [19].

Cinacalcet (Sensipar®/Mimpara®, Amgen Inc., Thousand Oaks, CA) is a first in-class calcimimetic that is indicated for the treatment of SHPT in adult subjects with CKD receiving dialysis [21]. The safety and efficacy of cinacalcet in adults with CKD and SHPT receiving dialysis has been extensively investigated [22–25], and its use, along with calcitriol, or vitamin D analogs, or a combination thereof, is included in the Kidney Disease Improving Global Outcomes (KDIGO) clinical practice guidelines for PTH lowering in adult subjects with stage 5 CKD receiving dialysis [1]. While calcimimetics have been shown to effectively control biochemical parameters in adult ESRD subjects with associated SHPT receiving dialysis, and severe SHPT with hypercalcemia after renal transplantation [26], the safety and efficacy of these agents in children needs to be further investigated [10]. In addition, while single-dose safety, pharmacokinetic (PK), and pharmacodynamic (PD) data have been published for children > 6 years of age, similar data from younger children have not previously been reported [27–29].

The objectives of this study were to evaluate the safety, tolerability, PK, and PD effects of cinacalcet after a single dose to pediatric subjects aged 28 days to < 6 years with CKD receiving dialysis. In addition, the study evaluated the safety of a 0.25 mg/kg starting dose, in pediatric subjects under the age of 6 years.

## Methods

**Fig. 1:**
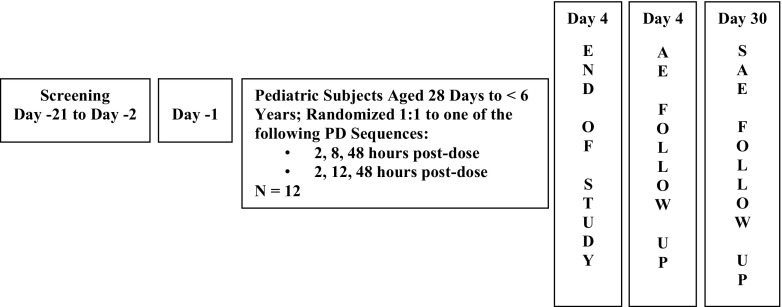
Study design and treatment schema. Screening was conducted between days − 21 to − 2. Subjects entered the clinical unit on day − 1 to undergo safety laboratory testing and baseline PD sampling and remained in residency until 24-h post-dose procedures were completed. Following pre-dose procedures and dosing on day 1, subjects underwent a 72-h period of PK, PD sampling, and safety monitoring. End of study procedures were conducted on day 4 (72 h post-dose). t SAE follow-up was conducted to day 30. *PD* pharmacodynamic, *PK* pharmacokinetic. *AE* adverse evemt. *SAE* serious adverse event

We conducted a phase 1, open-label study designed to evaluate the safety, tolerability, PK, and PD of cinacalcet in subjects aged 28 days to < 6 years with CKD and SHPT undergoing hemodialysis or peritoneal dialysis. The study was conducted in seven centers located in four countries. The first subject was enrolled on January 25, 2011, and the completion date was August 25, 2015 (Fig. 1).

Twelve subjects (< 6 years old) who met inclusion and exclusion criteria (Table 1) were enrolled to receive a single dose of 0.25 mg/kg cinacalcet. Cinacalcet was supplied as 5-mg capsules that were not to be swallowed whole. The capsules were opened and the contents mixed with purified water or USP-NF sucrose syrup and administered orally (syringe) or by nasogastric or gastric (NG/G) tube. If possible, subjects fasted from 2 h pre-dose to 2 h post-dose on day 1. If fasting was not possible, the subject’s intake of food (liquids and solids) was recorded in the electronic case report form (eCRF). For subjects undergoing dialysis on study day − 1 through day 4, information pertaining to the procedure (i.e., date (s), mode of dialysis, start and stop times, and dialysate composition [amount of calcium and potassium]) was recorded in the subject’s chart and in the appropriate eCRF. Based on the available data, half of the subjects received peritoneal dialysis and the other half received hemodialysis. It should be noted that hemodialysis or peritoneal dialysis does not impact cinacalcet PK or PD response [30]. All subjects received active vitamin D; two subjects (one in the 28 days to < 3 year age group and another in the ≥ 3 to < 6 year age group) received calcium-based phosphorus binders (Ca carbonate).

**Table 1:** Inclusion and exclusion criteria

**Key inclusion criteria**	
Age 28 days to < 6 years with CKD and sHPT, undergoing hemodialysis or peritoneal dialysis at screening (subjects ≥ 6 months should have been receiving dialysis for ≥ 1 month)	
Free of any disease or condition (other than those diseases or conditions related to their renal disease)	
Body weight ≥ 6 kg at screening and at day − 1; gestational age 30 weeks; physical examination must be acceptable to investigator at screening and at day − 1	
Serum calcium within age-appropriate normal ranges per NKF-K/DOQI guidelines at screening and at day − 1	
Hemoglobin ≥ 8 g/dL at screening and at day − 1	
Normal or clinically acceptable ECGs at screening and at day 1	
**Key exclusion criteria**	
Current or historic malignancy	
Cardiac ventricular arrhythmias within 28 days prior to screening	
A gastrointestinal disorder or surgery that could affect drug absorption (e.g., pyloric stenosis or any gut-shortening surgical procedure prior to screening)	
A new onset of seizure or worsening of a pre-existing seizure disorder within 2 months prior to IP administration	
Major surgery (defined as any surgical procedure that involves general anesthesia or respiratory assistance) within 28 days prior to screening	
Received therapy with cinacalcet within 1 month prior to randomization	
Clinical lab signs of hepatic impairment	
Medications: use of grapefruit juice, herbal medications, or potent CYP 3A4 inhibitors (e.g., erythromycin, clarithromycin, ketokonazole, itraconazole) within 14 days prior to enrollment and during study	
Concurrent or within 28 days prior to enrollment use of medications that are predominantly metabolized by the enzyme CYP2D6 with a narrow therapeutic index; use of medications that prolong QT interval	

*CKD* chronic kidney disease, *sHPT* secondary hyperparathyroidism, *NKF-KDOQI* National Kidney Foundation Kidney Disease Outcomes Quality Initiative, *ECG* electrocardiogram, *IP* intraperitoneal

Blood sampling at pre-defined time intervals post-dose was performed to assess cinacalcet pharmacokinetics and pharmacodynamics.

### Assessments

Blood samples were collected to measure PTH using an immunometric assay (ADVlA Centaur PTH Assay, Siemens Healthcare, Erlangen, Germany), total serum calcium, and albumin 1 day before (day − 1) cinacalcet was administered. Serum calcium was reported as total calcium or as the albumin-corrected value by the central laboratory based on calcium and albumin concentrations. In addition, ionized calcium was measured in whole blood samples to rapidly and efficiently monitor calcium status (e.g., normocalcemia, hypocalcemia). For all subjects, blood samples for PK and PD analyses were collected pre-dose. Subjects were randomized to undergo one of two sampling sequences post-dose: (1) sampling at 2, 8, and 48 h; or (2) sampling at 2, 12, and 48 h. Subjects were assessed clinically, including safety monitoring, for up to 72 h after dosing. Subjects were monitored carefully for the occurrence of hypocalcaemia (clinical symptoms, as well as serum calcium levels). In the event that serum calcium levels decreased below 8.4 mg/dL (2.1 mmol/L) and/or symptoms of hypocalcaemia occurred, the following options were recommended to investigators: calcium-containing phosphate binders, vitamin D sterols, and/or adjustment of dialysate calcium concentrations to raise serum calcium, according to clinical judgment. Serum phosphate was managed to achieve concentrations within age-appropriate ranges per National Kidney Foundation-Kidney Disease Outcomes Quality Initiative (NKF-K/DOQI) guidelines [1].

For determination of plasma cinacalcet concentrations, plasma samples were obtained and analysis was performed using high-performance liquid chromatography followed by tandem mass spectrometric detection (LC-MS/MS) at Covance (Madison, WI). The calibration standards and QC samples were interspersed with study samples within each analytical run, with a validated analytical range of 0.100 to 25 ng/mL. A total of 130 samples were analyzed and the overall precision and (mean) accuracy of the QC samples of all analytical runs were within the acceptance criteria. All study samples were analyzed within the known stability period of 347 days when stored at − 10 to − 30 °C. Pharmacokinetic (PK) analyses were conducted using Pharsight Knowledgebase Server system version 4.0.3 (Pharsight®, St. Louis, MO). Cinacalcet plasma concentration-time data was used to determine the PK parameters using non-compartmental methods (Phoenix WinNonlin v.6.4 software on Citrix [Pharsight®, St. Louis, Missouri]). Plasma concentrations below the lower limit of quantification (LLOQ; 0.100 ng/mL) were set to zero for the estimation of PK parameters for each subject and for the calculation of summary statistics at each time point. Actual dosing and sampling times were used for all calculations.

### Study endpoints

The primary endpoint of the study was the incidence of treatment-emergent adverse events (AEs), clinically significant changes in physical examinations, laboratory safety tests, electrocardiograms (ECG), and vital signs. The secondary endpoints included the PK parameters of cinacalcet (area under the plasma concentration-time curve (AUC), maximum observed plasma concentration (*C*_max_), time to *C*_max_ [*t*_max_], and half-life [*t*_1/2_]) and PD parameters of PTH and serum levels of calcium (total calcium, albumin corrected serum calcium [cCa], and ionized calcium). Safety endpoints included nature, frequency, severity, and relationship to treatment of all AEs; incidence of hypocalcemia; and vital signs and changes in laboratory parameters, including clinical chemistry.

### Statistical analysis and determination of sample size

The sample size was based on the variability of change from baseline in serum calcium in response to cinacalcet administered to pediatric patients 6 to < 18 years (Amgen Study 20030227) throughout the efficacy assessment phase (EAP), weeks 17–20. A power analysis of data collected from 12 subjects (standard deviation = 0.53 mg/dL) was calculated to have 80% power to detect a 0.48 mg/dL reduction in serum calcium from baseline (*α* = 0.05, two-sided). Descriptive statistics were provided for selected demographic, safety, PK, and PD data for all subjects. Descriptive statistics on continuous data included means, medians, standard deviations, and ranges, while categorical data were summarized using frequency counts and percentages.

### Safety monitoring and review

Treatment emergent AEs were grouped by system organ class and by preferred term within system organ class according to the Medical Dictionary for Regulatory Activities (MedDRA) AE-preferred term dictionary version 18.0. Determination of the severity of all AEs was consistent with Common Terminology Criteria for Adverse Events (CTCAE) V. 4.0 unless specified otherwise. The number and percent of subjects reporting at least one treatment-emergent AE and each treatment-emergent AE were summarized by system organ class and preferred term over all subjects and further classified by relationship to treatment. Tables of “on-study” deaths, serious AEs, and early withdrawals due to AEs were provided when necessary.

## Results

### Demographic and baseline characteristics

Fourteen subjects were initially enrolled into the study including five subjects 28 days to < 3 years of age and nine subjects ≥ 3 years to < 6 years of age. One subject in each age group discontinued the study prior to receiving cinacalcet. Thus, four subjects in the original 28 days to < 3 years age group and eight subjects in the ≥ 3 years to < 6 years age group completed the study. Among those patients who completed the study, the mean age was 18.8 months (range 8 to 34 months) for the younger group subjects (< 3 years), 51.5 months (range 44 to 61 months) for subjects ≥ 3 years to < 6 years, and 40.6 months (range 8 to 61 months) for all subjects. Mean baseline concentrations of PTH, corrected calcium, and phosphorus were 536.2 pg/mL, 2.6 mmol/L, and 1.8 mmol/L, respectively; the mean baseline Ca × P product was 58.6 mg^2^/dL^2^.

### Primary endpoint

All subjects were monitored for adverse events following a single oral dose of 0.25 mg/kg cinacalcet. Five treatment-emergent AEs were reported for 3 (25%) of 12 subjects, including 1 (25%) of 4 subjects < 3 years old, and 2 (25%) of 8 subjects ≥ 3 years to < 6 years old. There were no serious or fatal AEs. Adverse events reported for one subject (34 months old) were vomiting, catheter site hemorrhage, and catheter expulsion. Adverse events reported for two subjects ≥ 3 years to < 6 years old were increased body temperature (age 61 months) and hypocalcemia (treatment-related adverse event of interest, age 45 months).

There were no life-threatening (grade 4) events reported during the study. Severe (grade 3) events included one event each of catheter site hemorrhage, device expulsion (chest catheter falling out), and hypocalcemia (asymptomatic, non-serious) that resolved without treatment on day 3 after start of the treatment. The patient who experienced asymptomatic hypocalcemia (non-serious adverse event, CTCAE grade 3) on day 1, hour 8 after cinacalcet administration was a female with baseline total Ca = 2.1 mmol/L; albumin corrected Ca (cCa) = 2.2 mmol/L, ionized Ca = 0.9 mmol/L (in the normal range for pediatric subjects) with an elevated baseline PTH of 126.4 pmol/L (1191 pg/mL), and a phosphorus = 1.71 mmol/L. The patient was undergoing hemodialysis with a dialysate calcium content of 3 mg/dL and received active vitamin D, Ca carbonate, antihypertensives (amlodipine, isradipine, labetalol), sevelamer, docusate, sodium polystyrene sulfonate (K binder), and pyridoxine. Changes observed in laboratory test results (e.g., chemistry and hematology data) were consistent with the disease states under study. No notable changes from baseline were observed for vital signs measurements or ECG results.

### Secondary endpoints

#### Pharmacokinetics

**Fig. 2:**
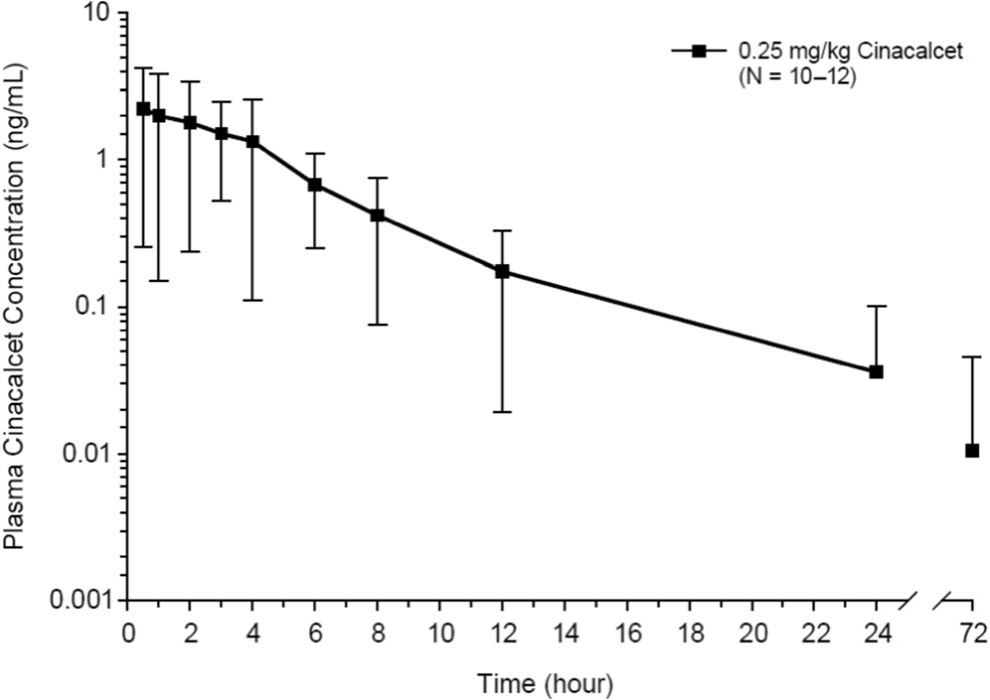
Mean (SD) plasma cinacalcet log concentration-time profiles after administration of cinacalcet at 0.25 mg/kg to pediatric subjects with chronic kidney disease receiving dialysis (*n* = 10–12)

**Fig. 3:**
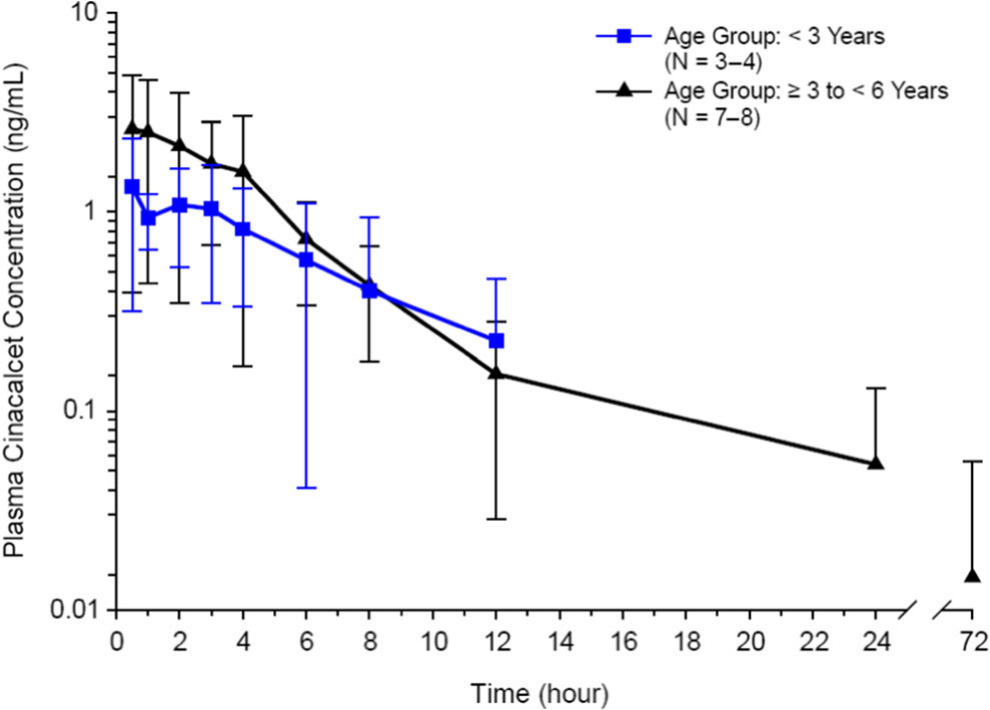
Mean (SD) plasma cinacalcet log concentration-time profiles by age group after enteral administration of cinacalcet

Cinacalcet was rapidly absorbed, with a median *t*_max_ of 1 h (range 0.50 to 4.0 h) (Table 2, Fig. 2). The mean (SD) half-life (*t*_½,z_) was 3.70 (2.57) h (Table 2). Plasma cinacalcet mean (SD) *C*_max_ and AUC_last_ in all subjects < 6 years of age were 2.83 (1.98) ng/mL and 11.8 (8.74) h*ng/mL, respectively (Table 2). While overall there was no clinically meaningful difference in cinacalcet exposure between the age groups due to extensive overlap and high inter-subject variability (coefficient of variation [CV%] range 54.5 to 75.7) (Fig. 3), there was a trend for slightly higher exposures in subjects ≥ 3 to < 6 years old compared to subjects < 3 years old. Mean plasma cinacalcet *C*_max_ and AUC values appeared to be marginally higher (1.6- to 2.3-fold) in subjects ≥ 3 to < 6 years old compared with subjects < 3 years old, but individual values were within a similar range. There were no notable effects of age, weight, body surface area, and BMI on the PK of cinacalcet, based on an assessment of the impact of these characteristics on cinacalcet *C*_max_ and AUC values.

**Table 2:** Pharmacokinetic parameter estimates for cinacalcet in plasma after administration of 0.25 mg/kg cinacalcet to pediatric subjects < 6 years old with CKD receiving dialysis

Parameter	*t*_max_ (h)	*C*_max_ (ng/mL)	AUC_last_ (h*ng/mL)	AUC_inf_ (h*ng/mL)	*t*_1/2,z_ (h)
**Subjects < 3 years old**					
*N*	4	4	4	4	4
Mean (SD)	NR	1.51 (0.820)	7.21 (5.27)	8.31 (6.28)	2.73 (0.952)
Median	0.75	1.36	6.04	6.68	2.60
Min–max	0.50–3.1	0.797–2.51	2.84–13.9	3.29–16.6	1.83–3.87
CV%	NR	54.5	73.1	75.7	34.9
**Subjects ≥ 3 to < 6 years old**					
*N*	8	8	8	7	7
Mean (SD)	NR	3.50 (2.09)	14.1 (9.49)	12.9 (8.60)	4.26 (3.09)
Median	1.0	3.97	13.7	9.66	2.95
Min–max	0.50–4.0	0.818–5.75	3.52–28.6	3.90–25.4	2.06–10.6
CV%	NR	59.9	67.3	66.5	72.6
**All subjects < 6 years old**					
*N*	12	12	12	11	11
Mean (SD)	NR	2.83 (1.98)	11.8 (8.74)	11.3 (7.86)	3.70 (2.57)
Median	1.0	2.18	8.96	9.66	2.95
Min–max	0.50–4.0	0.797–5.75	2.84–28.6	3.29–25.4	1.83–10.6
CV%	NR	70.0	74.1	69.8	69.4

*AUC* area under the plasma-concentration-time curve, *AUC*_*inf*_ AUC from time zero to infinity, *AUC*_*las*t_ AUC from time zero to time of last quantifiable concentration, *CKD* chronic kidney disease, *C*_*max*_ maximum observed plasma concentration, *CV%* coefficient of variation, *max* maximum, *min* minimum, *NR* not reported, *SD* standard deviation, *t*_*1/2,z*_ terminal half-life associated with *λ*_z_, *t*_*max*_ time to maximum concentration

#### Pharmacodynamics

**Fig. 4:**
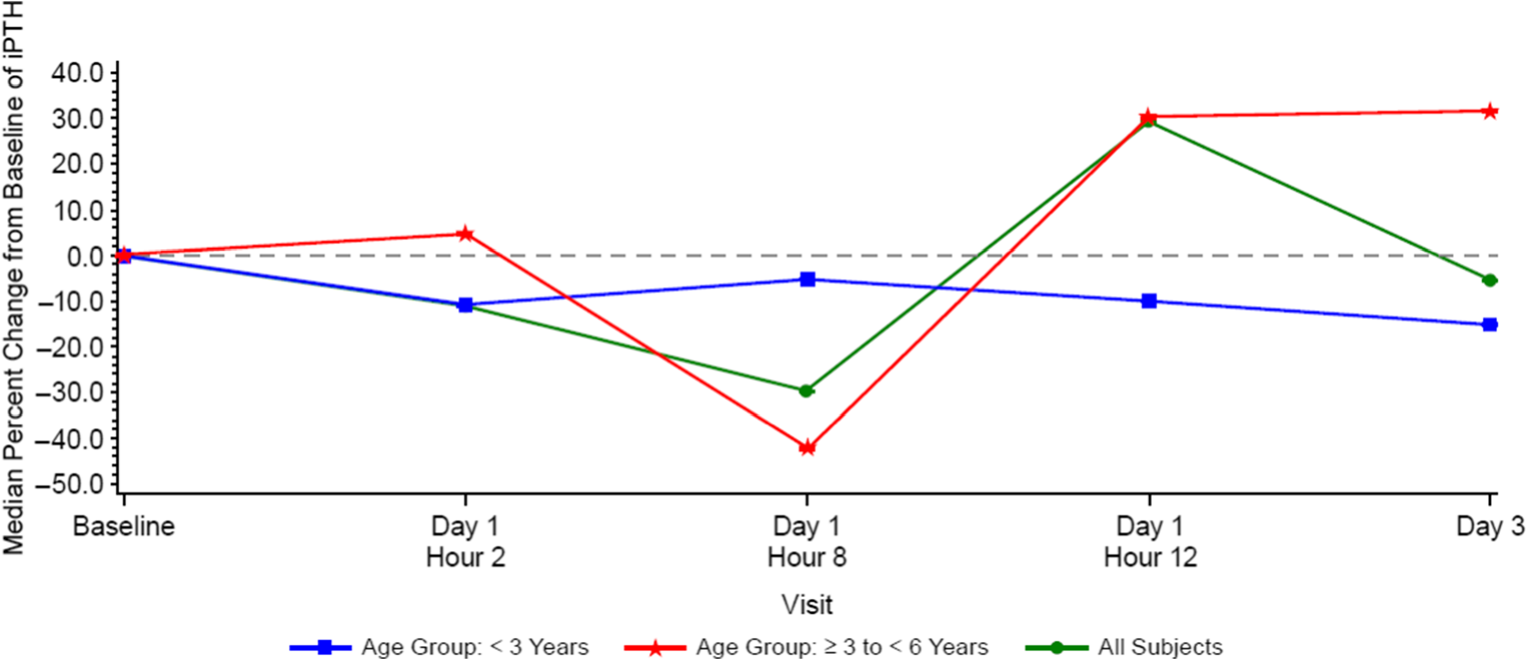
Median parathyroid hormone (PTH) percent change from baseline after administration of cinacalcet at 0.25 mg/kg to pediatric subjects with chronic kidney disease (CKD) receiving dialysis

**Fig. 5:**
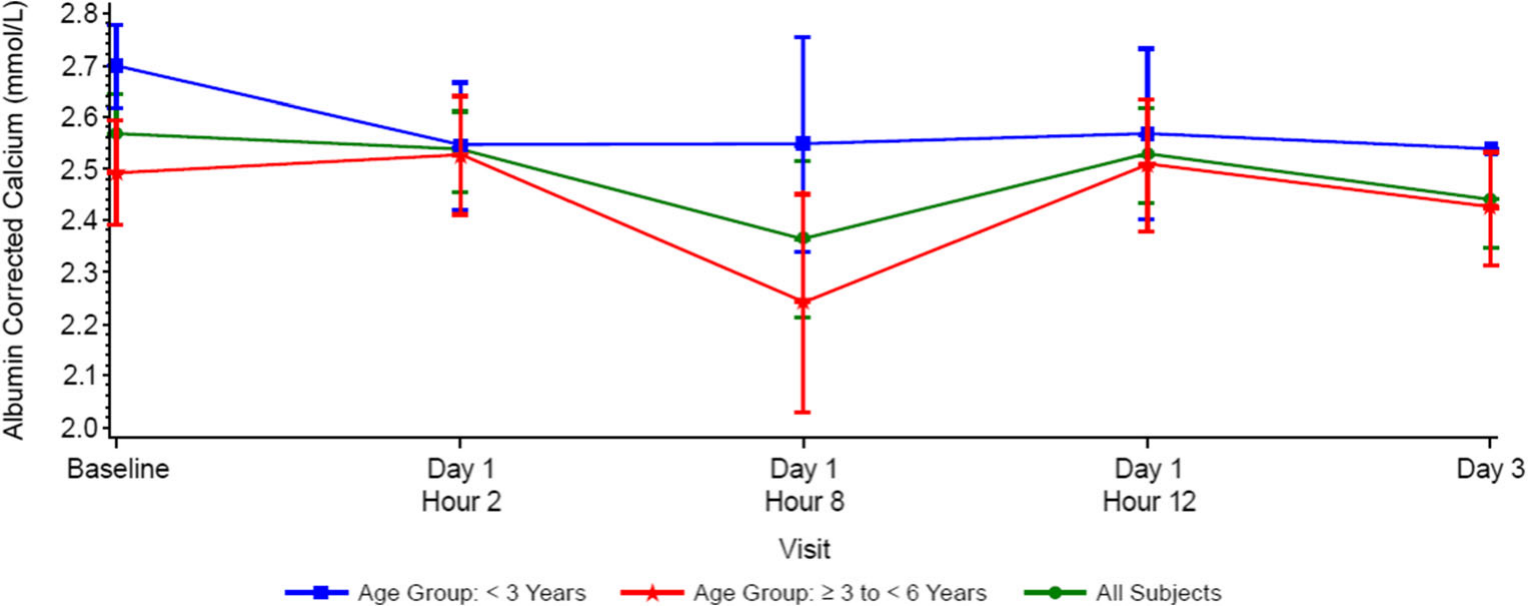
Mean (SE) albumin corrected calcium (cCa) over time by age group (< 3 years (*n* = 4); age ≥ 3 years < 6 years (*n* = 8); all subjects (*n* = 12))

**Fig. 6:**
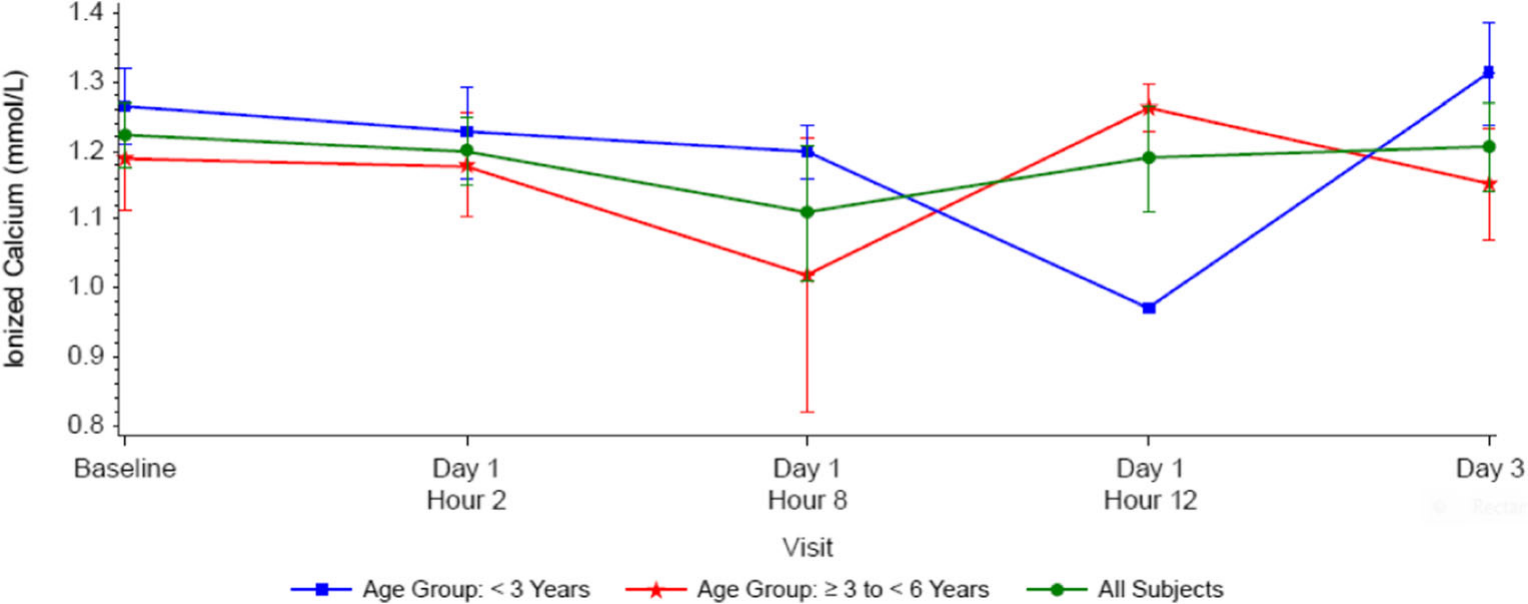
Mean (SE) ionized calcium (cCa) concentrations over time by age group (< 3 years (*n* = 4); age ≥ 3 years < 6 years (*n* = 8); all subjects (*n* = 12))

Baseline PTH values varied widely (10.5–1373.6 pg/mL; median [Q1, Q3] 161 pg/mL [123 pg/mL, 1263 pg/mL]), as there were no inclusion criteria for minimum PTH levels. Reductions in serum PTH from baseline were observed at 2 to 8 h post-dose (median 10.8% and 29.6%, respectively) and returned towards baseline by 12 to 72 h (Fig. 4 or Table 3). Median percent reductions in PTH were greater at 8 h post-dose in subjects ≥ 3 years to < 6 years old (− 42.2%) than in subjects < 3 years old (− 5.2%); however, this observation should be interpreted with caution given the small subject numbers (*N* = 3 and *N* = 2, respectively) (Fig. 4 or Table 3). Albumin-corrected serum calcium levels decreased slightly from baseline, reached nadirs at 8 h post-dose, and subsequently returned to baseline (Fig. 5). Serum ionized calcium levels also decreased slightly from baseline, reached nadirs at 8 h post-dose in subjects ≥ 3 years to < 6 years old, at 12 h post-dose in subjects < 3 years old and subsequently returned to baseline (Fig. 6).

**Table 3:** Serum parathyroid hormone (pg/mL) at baseline and after administration of cinacalcet

	Age group: < 3 years (*N* = 4)	Age group: ≥ 3 years to < 6 years (*N* = 8)	Total subjects (*N* = 12)
**Baseline**			
*n*	4	8	12
Median	268.8	160.8	160.8
Q1, Q3	125.1, 869.7	102.4, 1266.8	122.6, 1262.6
Min, max	119.1, 1333.0	10.5, 1373.6	10.5, 1373.6
**Day 1, hour 2 (% change)**			
*n*	3	6	9
Median	− 10.8	4.8	− 10.8
Q1, Q3	− 18.5, 19.1	− 53.3, 53.0	− 18.5, 23.1
Min, max	− 18.5, 19.1	− 89.0, 6253.2	− 89.0, 6253.2
Day 1 hour 2 (median)	331.3	135.6	156.2
**Day 1 hour 8 (% change)**			
*n*	2	3	5
Median	− 5.2	− 42.2	− 29.6
Q1, Q3	− 12.6, 2.3	− 54.8, − 29.6	− 42.2, − 12.6
Min, max	− 12.6, 2.3	− 54.8, − 29.6	− 54.8, 2.3
Day 1, hour 8 (median)	244.8	620.8	355.37
**Day 1, hour 12 (% change)**			
*n*	1	4	5
Median	− 10.0	30.3	29.4
Q1, Q3	− 10.0, − 10.0	28.5, 9471.7	27.7, 31.2
Min, max	− 10.0, − 10.0	27.7, 18,912.2	− 10.0, 18,912.2
Day 1, hour 12 (median)	1199.1	167.1	171.1
**Day 3 (% change)**			
*n*	3	6	9
Median	− 15.0	31.8	− 5.4
Q1, Q3	− 42.3, 8.4	− 42.9, 83.8	− 42.3, 69.0
Min, max	− 42.3, 8.4	− 49.2, 16,816.6	− 49.2, 16,816.6
Day 3 (median)	345.4	515.6	345.4

*N* number of subjects in the analysis set, *n* number of subjects with non-missing data at the time point of interest

#### Pharmacokinetic/pharmacodynamic relationships

**Fig. 7:**
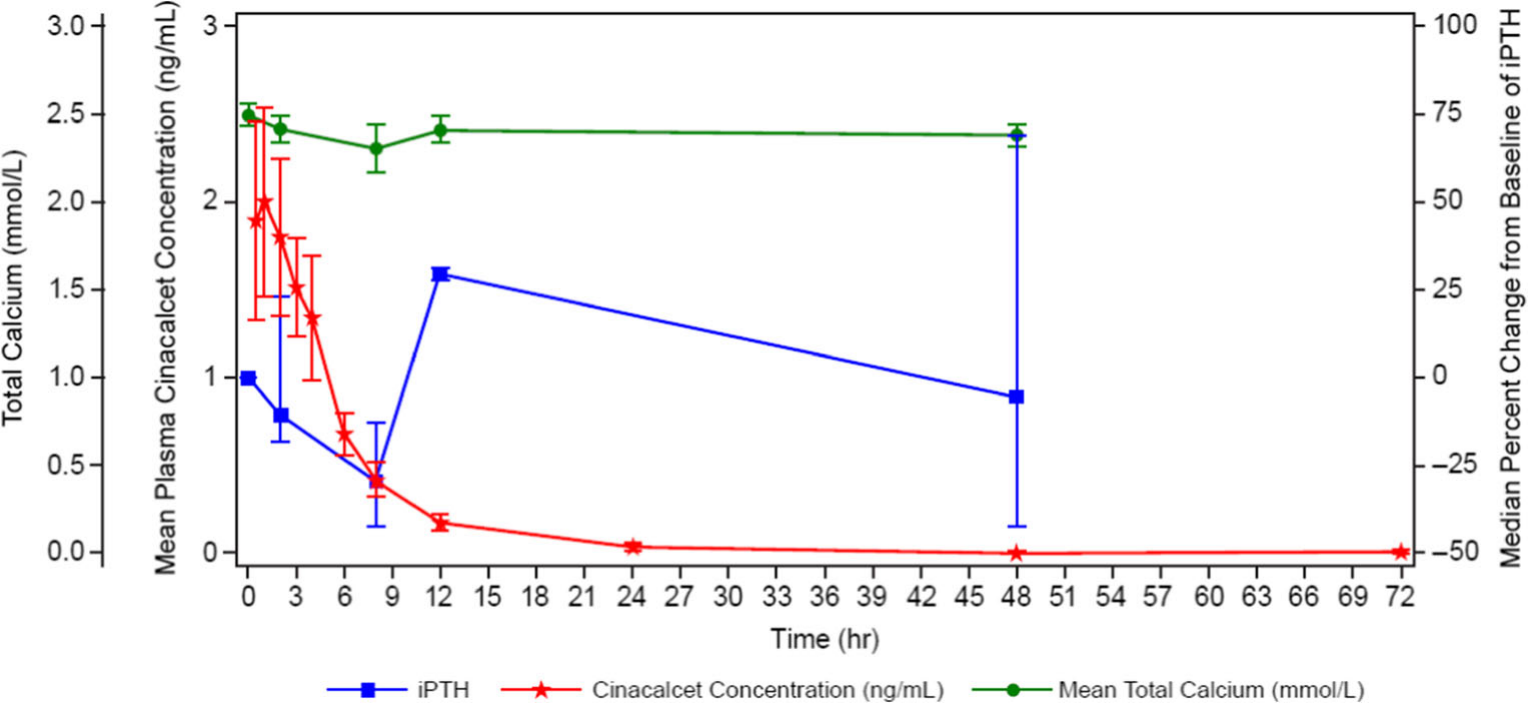
Mean (SE) plasma cinacalcet, median (Q1, Q3) parathyroid hormone (PTH) percent change from baseline; mean (SE) total serum Ca concentration-time profiles in pediatric subjects with chronic kidney disease (CKD) receiving dialysis

Median percent reductions in plasma PTH concentration were associated with increases in cinacalcet concentration immediately after dosing (Fig. 7). Specifically, mean cinacalcet plasma concentration peaked at 1 h post-dose and gradually declined to undetectable levels by day 2. As cinacalcet concentrations declined, plasma PTH concentrations transiently increased and returned to baseline levels by day 3. As expected, slight reductions in total serum calcium (median percent reductions of up to 6.1% at 12 h post-dose) were observed and were followed by a return to baseline by the end of study. The overall pattern of changes from baseline in mean serum calcium and PTH concentrations was similar for both age groups. Decreased serum calcium and PTH corresponded inversely to changes in cinacalcet plasma concentrations up to 8 h after administration. As cinacalcet concentrations declined towards lower levels (< 0.5 ng/mL), PTH and serum calcium increased and returned to baseline levels by day 3 (Fig. 7).

#### Safety

No major or unexpected safety findings were identified in this study [27]. Three subjects (25%) experienced non-serious AEs (one subject had asymptomatic hypocalcemia, one subject had increased body temperature, and one subject experienced vomiting, catheter site hemorrhage, and expulsion). Overall, no clinically significant changes were observed in vital signs, hematology parameters, or clinical chemistry parameters, with the exception of median PTH and calcium concentrations.

## Discussion

The present study evaluated the safety of cinacalcet, including the incidence of treatment-emergent AEs and clinically significant changes in physical examinations, laboratory safety tests, electrocardiograms ECG, and vital signs in dialyzed children < 6 years of age. No subject experienced a serious AE, and three subjects experienced non-serious AEs, including hypocalcemia, body temperature increase, and vomiting. No additional safety findings were identified.

We found that the pharmacokinetic parameters for cinacalcet in pediatric subjects ≤ 6 years of age were similar to those observed in other studies (unpublished observations) when normalized by dose and body weight. Overall, no clinically meaningful differences in cinacalcet exposure were observed between the age groups due to extensive overlap, high inter-subject variability, and low plasma levels from 12 to 72 h after dosing (Table 2; Fig. 3). However, cinacalcet exposure levels (*C*_max_ and AUC values) tended to be higher in subjects ≥ 3 to < 6 years old than in subjects < 3 years old. Specifically, mean plasma cinacalcet *C*_max_ and AUC values were 1.6- to 2.3-fold higher in subjects ≥ 3 to < 6 years old compared with subjects < 3 years old. Consequently, mean *C*_max_ (ng/mL) concentrations for the younger age group was ~ 57% lower than the older age group subjects and AUC_last_ was ~ 49% lower. The trend for higher cinacalcet exposure levels in the older age group is more apparent when examined within the first 4 h after dosing. Although sample sizes are small, one possible reason explaining this trend may be a transient and heightened increase in CYP3A4 expression at or above mature levels within 1 to 2 years immediately following birth that would result in similar or greater metabolism of cinacalcet and consequently lower AUC and *C*_max_ levels in the younger population [31]. Another possible explanation of the lower cinacalcet exposure levels in younger children is that they have a higher proportion of total body water [32] and thus, a greater volume of distribution of cinacalcet, resulting in lower measured plasma concentrations. In agreement with other pediatric studies, there were no notable effects of age, weight, body surface area, and BMI on the PK of cinacalcet (unpublished observations).

The 0.25 mg/kg dose employed had an observable PD effect as demonstrated by the lowering of PTH levels from baseline after cinacalcet administration, with a maximum reduction observed at 8 h post-dose. PTH levels decreased and were associated inversely with increases in plasma cinacalcet concentrations. A similar, but milder, inverse relationship between serum calcium and increased plasma cinacalcet concentrations was observed; however, only one subject had asymptomatic hypocalcemia. These results are consistent with the PD response and safety profile for cinacalcet in adults and children 6 to 18 years of age [27, 29, 33].

The dose used in the current study is approximately half the adult starting dose based on a milligrams per kilogram basis and is lower than the mean dose of 0.39 mg/kg previously studied in 12 subjects age 6 to < 18 years with CKD receiving dialysis, where 2 subjects (17%) experienced AEs (unpublished observations). In the current study, five treatment-emergent AEs were reported for 3 (25%) of 12 subjects, including 1 (25%) of 4 subjects < 3 years old, and 2 (25%) of 8 subjects ≥ 3 years to < 6 years old, and all were mild to moderate in severity. The safety profile and PD effect observed in the present study are similar to those observed in a previous cohort of older pediatric subjects with sHPT on dialysis [27–29].

The main limitation of the current study was the small sample size, which did not permit extensive subgroup analyses by age, and therefore, no formal statistical testing was performed. However, the results from this single-dose study provide valuable safety, pharmacokinetic, and pharmacodynamic information in a particularly young cohort of pediatric dialysis patients with SHPT.

## Conclusions

The observed safety profile and PD response in this study of cinacalcet in pediatric dialysis patients less than 6 years of age were consistent with that known for adult subjects with SHPT and in older pediatric dialysis subjects 6 to < 18 years of age with SHPT. Results from this study demonstrate that a 0.25 mg/kg dose of cinacalcet was found to be safe and well-tolerated in children under 6 years of age, and the observed PD response suggests that repeated doses would result in a clinically meaningful decrease in PTH levels.
